# Case Report: A case of *Chlamydia psittaci* infection in an HIV patient

**DOI:** 10.3389/fcimb.2023.1185803

**Published:** 2023-05-16

**Authors:** Wenwu Yao, Xuhui Yang, Jinchuan Shi, Zhangnv Yang, Ying Yao, Jun Kou, Shelan Liu, Linbo Wang, Zhuoyin Wu, Guoxiang Shi, Hao Yan, Yajun Song

**Affiliations:** ^1^ Department of Microbiology and Department of Infectious Diseases, Zhejiang Provincial Center for Disease Control and Prevention, Hangzhou, China; ^2^ State Key Laboratory of Pathogen and Biosecurity, Beijing Institute of Microbiology and Epidemiology, Beijing, China; ^3^ Department of Health Monitoring, Hangzhou Center for Disease Control and Prevention, Hangzhou, China; ^4^ Affiliated Hangzhou Xixi Hospital, Zhejiang University School of Medicine, Hangzhou, China; ^5^ Department of Infectious Diseases, Disease Prevention Control Center of Shangcheng District, Hangzhou, China

**Keywords:** *Chlamydia psittaci*, psittacosis, next-generation sequencing, diagnosis, genotype

## Abstract

*Chlamydia psittaci* is the pathogen of psittacosis and infects a wide range of birds and even humans. Human infection occurs most commonly in those with a history of contact with birds or poultry. We describe a case of psittacosis in a human immunodeficiency virus infected patient in Zhejiang Province for the first time. *C. psittaci* infection was confirmed by nested polymerase chain reaction (PCR) and Real-Time PCR. Phylogenetic analysis revealed that the sequences from the patient’s samples clustered with genotype A in the same branch. Our study highlights the possibility of diagnosing psittacosis in patients with a chronic disease such as HIV-infected patients, and should increase awareness and surveillance of psittacosis in China.

## 1 Introduction

Human psittacosis, also known as parrot fever or ornithosis, is a zoonotic infectious disease whose agent is the obligate intracellular bacterium *Chlamydia psittaci*. Although psittacosis is not a common disease, it has been reported worldwide, including in China, the USA, Europe, and Australia (Yung and Grayson, 1988; Branley et al., 2014; Shaw et al., 2019). It accounts for 1%–2% of cases of community-acquired pneumonia (CAP) annually (Hogerwerf et al., 2017). However, the clinical manifestations vary from asymptomatic infection to fatal systemic illness (Crosse, 1990; Branley et al., 2014).

There are several techniques to identify *C. psittaci*, including serological techniques, isolation, direct immunoenzymatic tests and polymerase chain reaction (PCR) (Mahony et al., 1993; Longbottom and Coulter, 2003; Jeong et al., 2017). Metagenomic next-generation sequencing (mNGS) is a new technique that has been widely used in the diagnosis of pathogens in patient infected by human immunodeficiency virus (Zhu et al., 2022), especially when clinicians cannot determine the pathogen causing the disease (Gu et al., 2019). In recent years, with improvements in detection methods and understanding of the disease, reported cases of psittacosis have been increasing.

Here, we present a successfully cured case of psittacosis infection in an HIV patient diagnosed by mNGS, nested PCR and Real-Time PCR. To the best of our knowledge, this is the first case of infection caused by *C. psittaci* in an HIV patient in Zhejiang Province.

## 2 Case presentation

The patient was a 65-year-old man who had suffered from HIV for 13 years. He was admitted to the hospital on April 6, 2022 with chills and fever after visiting the cemetery on Chinese Tomb Sweeping Day. The highest recorded body temperature was 39.9°C and was accompanied by cough and expectoration, white sticky sputum, no abdominal distension or abdominal pain, pain in the joints and muscles of the body, no loss of smell or taste, no headache or dizziness, no frequent urination or urgency, no diarrhea and no chest pain or hemoptysis.

Chest computed tomography (CT) suggested that lung infection foci should be considered on both sides. Therefore, “community acquired pneumonia and AIDS” were diagnosed, and the patient was admitted to the second hospital of Haishu District, Ningbo City, China (April 9–16, 2022).

After the patient was initially infected with HIV 13 years ago, he was regularly treated with lamivudine, zidovudine and nevirapine. Viral load was frequently below the detection limit. However, recently, CD^4+^ T cell counts were greater than 300/UL in a recent routine blood examination. After admission, butravil was prescribed as a replacement to continue antiviral treatment.

Blood tests and cultures of anaerobic bacteria were negative, and sputum acid fast bacillus smears were negative. Mycoplasma pneumoniae antibody IgM was positive. Piperacillin/tazobactam, imipenem/cilastatin sodium (from 4 to 11) and voriconazole were given as treatment, but his condition did not improve, so the patient came to Hangzhou Xixi hospital for further diagnosis and treatment. Bronchoalveolar lavage fluid (BALF) was used to perform mNGS, and the results revealed 1082 sequence reads corresponding to *C. psittaci*. No sequence reads corresponded to other pathogenic microbes. *C. psittaci* pneumonia was confirmed by nested PCR and qPCR at the Zhejiang Provincial Center for Disease Control and Prevention (CDC).

**Figure 1:**
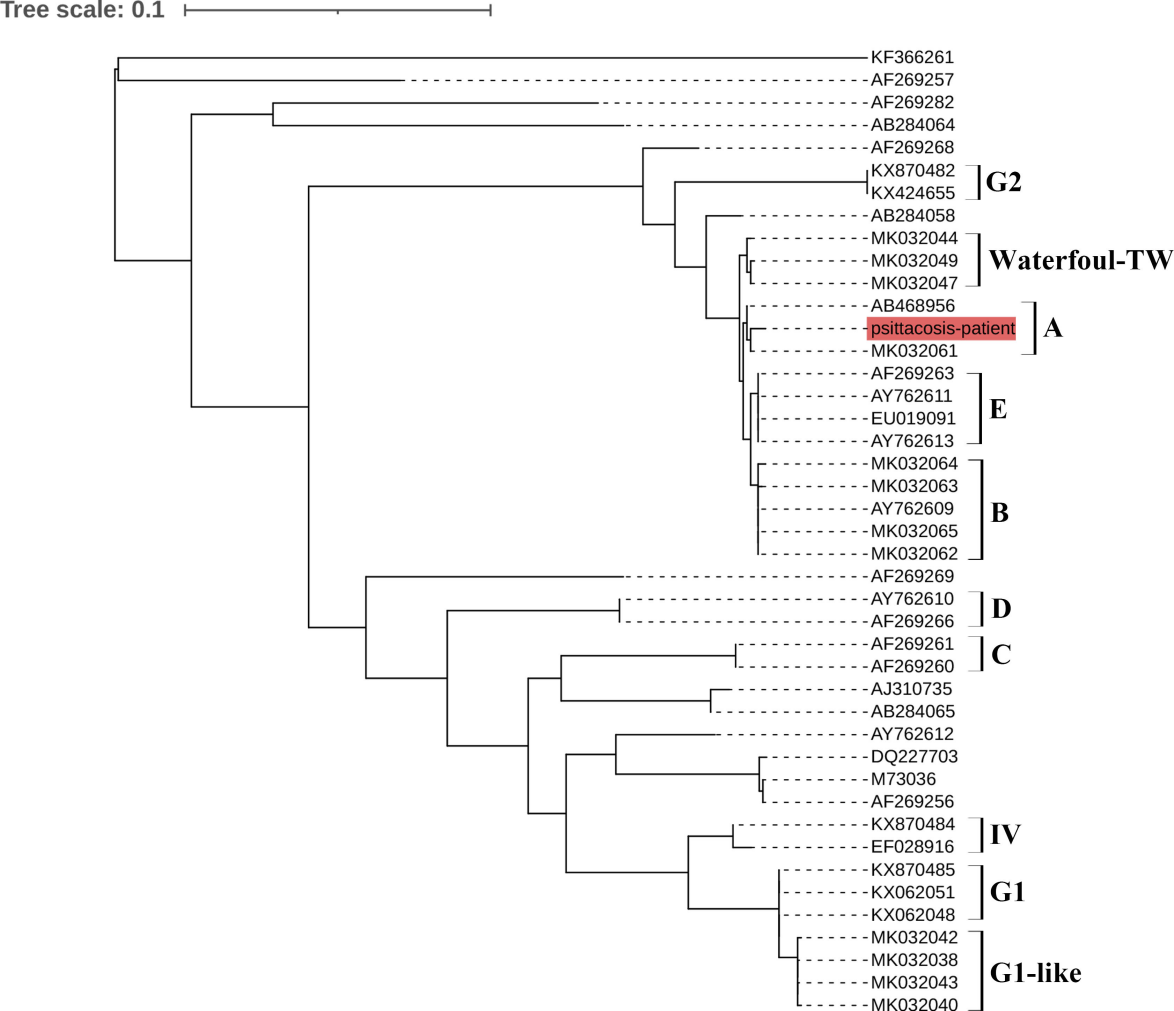
Phylogenetic tree of *C. psittaci* sequences from an HIV patient. The psittacosis-patient sequence from the patient is shown in red. A–E, G1, G2, waterfowl-TW and IV indicate different genotypes.

The extracted DNA was detected using real-time qPCR targeting the *C. psittaci* 16S rRNA locus tag and nested PCR targeting the major outer membrane protein (ompA) gene as described previous (Yao et al., 2022). Briefly, the total DNA of the samples was extracted using a commercial kit (Qiagen, USA), according to the manufacturer’s instructions. The real-time PCR was performed with the following steps: denaturation at 95°C for 30 s, 45 cycles of denaturation step at 94°C for 10 s, annealing at 60°C for 30 s. The nested PCR (first round) was performed with 1 cycle of 95°C for 2 min, followed by 35 cycles of 95°C for 1 min, 55°C for 30 s, and 72°C for 1 min, and a final extension at 72°C for 10 min, second round cycling conditions were 1 cycle of 95°C for 2 min, followed by 35 cycles of 95°C for 1 min, 50°C for 30 s, and 72°C for 1 min, and a final extension at 72°C for 10 min. The products of nested PCR were sequenced at a commercial company (Sangon, China). The sequences were analyzed by using MEGA version 6.06, bootstrap values ≥ 70% were calculated from 1000 replicates and the phylogenetic tree was obtained. The phylogenetic tree showed that the sequence (named psittacosis-patient) from the patient’s samples clustered with MK032061 Taiwan/2018 and AB468956 Japan/2005 in the same branch (**Figure 1**). MK032061 and AB468956 are both classified as genotype A.

After the patient was diagnosed with *C. psittaci* infection, the antibacterial agents were switched to moxifloxacin and azithromycin. The patient’s condition improved significantly, PCR tests of the patient’s throat swab were negative for *C. psittac* on April 20, 2022 and he was discharged from the hospital on May 15, 2022.

## 3 Discussion

In this study, we describe a case of psittacosis in a patient who had suffered from HIV for 13 years. The patient was first admitted to the hospital with fever and chills. The chest CT suggested lung infection, which doctors considered to be a complication of AIDS. As the fever persisted, mNGS was applied to reveal the infectious pathogen. The results of mNGS suggested the possibility of *C. psittaci* infection. Later epidemiological investigations revealed that the patient had kept parrots in his house before the onset of the disease. Laboratory investigations were then initiated to establish a diagnosis for *C. psittaci* infection. The case was confirmed by qPCR and nested PCR. Appropriate treatment was initiated, which aided the full recovery of the patient.

Psittacosis most commonly occurs in people who work in close contact with birds or poultry, either in occupational settings or through companion bird exposure (Gaede et al., 2008; Harkinezhad et al., 2009). The global prevalence of *C. psittaci* in birds is estimated to be around 20%, and its transmission to humans has been reported regularly. Zhejiang Province is home to a range of wild bird hosts, and many families in this rural area raise poultry. These factors increase the risk of human infection with *C. psittaci*. Although an increase in reported cases has been observed, psittacosis is still regarded as an uncommon disease. Therefore, it is necessary to discuss and draw attention to clinical awareness and surveillance of the disease.

However, psittacosis lacks specific clinical manifestations and tends to cause similar clinical symptoms to other pathogens. This makes it difficult to diagnose, especially in those with chronic diseases. Hence, a diagnosis of psittacosis should be considered when there is a clear history of poultry or bird exposure and community-acquired pneumonia.

mNGS is a new tool that is rapid and accurate. It has been used in the diagnosis of viruses, bacteria, fungi, parasites and mycoplasma, and there have been several recent reports of parasites detected using mNGS (Schlaberg et al., 2017). However, mNGS should be used as a detection aid but not a gold standard for psittacosis detection, as the results of mNGS can indicate several pathogens. Therefore, for pathogen identification, mNGS needs to be combined with clinical tests. For the patient in our study, mNGS suggested that the *C. psittaci* gene was present in the patient’s BALF, so samples were sent to the Zhejiang CDC for identification by both qPCR and nested PCR. Compared to previous reports, which used only mNGS to detect psittacosis (Gu et al., 2020; Shi et al., 2021; Yuan et al., 2021), our experiments provide strong evidence of the presence of *C. psittaci* in this patient.

Finally, genotyping revealed that the patient was infected with *C. psittaci* genotype A. Each genotype tends to be associated with particular hosts; for example, genotypes A and B are associated with psittacine birds and pigeons, respectively. Genotype C is usually isolated from waterfowl and genotype D from turkeys (Harkinezhad et al., 2009). In our study, phylogenetic analysis showed that the psittacosis-patient sequence belonged to genotype A. The results of detection combined with the epidemiological findings suggested that this patient was likely to have been infected by a parrot. Unfortunately, the parrot was released after the patient became ill, so we could not obtain a sample from the parrot, and the status of the bird remains unknown. This is one drawback of our study.

In conclusion, we present the first instance of detection of *C. psittaci* infection in an HIV patient in China using mNGS and confirmation of the case using qPCR and nested PCR. Our study highlights that people with chronic disease handle domestic animals or wild birds should be alert for psittacosis. Drawing attention to the risk of psittacosis for people with chronic diseases is essential, and health administrations should increase awareness and surveillance of psittacosis in China.

## Data availability statement

The original contributions presented in the study are included in the article/supplementary materials, further inquiries can be directed to the corresponding author/s.

## Ethics statement

This study was approved by the Institutional Ethics Committee of the Zhejiang Provincial Center for Disease Control and Prevention. Written informed consent was obtained from the patient involved in this study. Written informed consent was obtained from the participant/patient(s) for the publication of this case report.

## Author contributions

WY and XY have contributed to original draft preparation of this manuscript. JS, ZY, YY, and JK have contributed to the investigation. LW, ZW, and GS review and edited the manuscript. HY and YS contributed to the project administration. All authors contributed to the article and approved the submitted version.
